# Supplementary material for: Cosmc controls B cell homing
Source: Nat Commun. 2020 Aug 10;11:3990. doi: 10.1038/s41467-020-17765-6 (PMC7417590; doi:10.1038/s41467-020-17765-6)
Supplement: Supplementary file 1 — Supplementary Information [file 41467_2020_17765_MOESM1_ESM.pdf]

Supplementary Information

Cosmc Controls B Cell Homing

Junwei Zeng, Mahmoud Eljalby, Rajindra P. Aryal, Sylvain Lehoux, Kathrin Stavenhagen,
Matthew R. Kudelka, Yingchun Wang, Jianmei Wang,
Tongzhong Ju, Ulrich H. von Andrian, Richard D. Cummings

Supplementary Figures 1-9

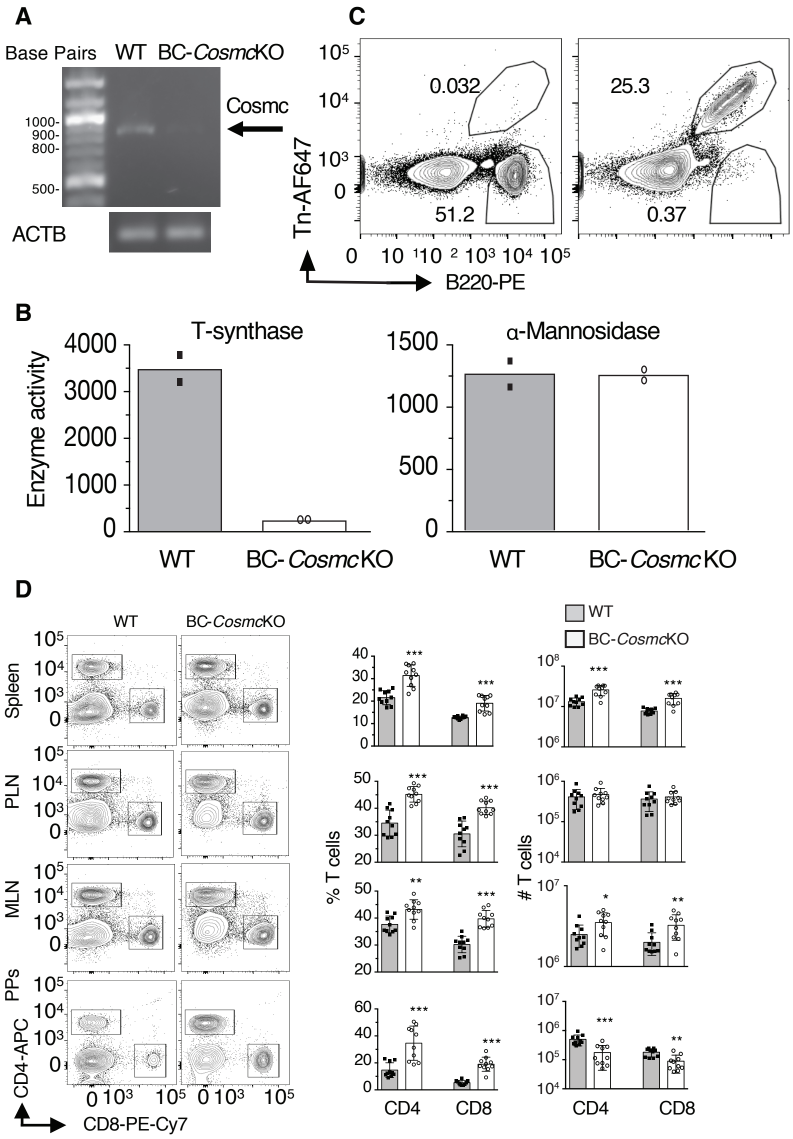

Supplementary Figure 1 Targeted deletion of *Cosmc* gene in B cells. (A) Semiquantitative RT-PCR of purified WT and BC-*Cosmc*KO B cells. A representative agarose gel image of PCR products from both genotypes (n=2) is presented. (B) T-synthase and α-Mannosidase activity of purified WT and BC-*Cosmc*KO B cells. T-synthase data are representative of two independent experiments, with two mice in each experiment (n=2), α-Mannosidase data are from two mice, and values are expressed as mean. (C) Representative flow cytometric dot plots showing anti-Tn staining of B cells, and T cell subsets of indicated organs (D) from WT (n=10) and BC-*Cosmc*KO (n=10) mice. For spleen, p values<0.0001 for both percentage and numbers of T cells; for PLN, p values<0.0001 for percentage of both CD4 and CD8 T cells, p value=0.5304 for CD4 and 0.5379 for CD8 T cells; for MLN, p values=0.0017 for percentage of CD4 T cells and <0.0001 for CD8 T cells, p value=0.0311 for the numbers of CD4 and 0.0066 for CD8 T cells; for PPs, p values=0.0002 for percentage of CD4 T cells and <0.0001 for CD8 T cells, p value=0.0005 for the numbers of CD4 and 0.0017 for CD8 T cells. Each symbol (black square and open circle for WT and BC-*Cosmc*KO, respectively) represents an individual mouse. Data are presented as average ±SD of each genotype. Unpaired two-tailed student t tests were performed to determine statistical significance with *** denoting p<0.001, ** p<0.01, * p<0.05. Source data are provided as a Source Data file.

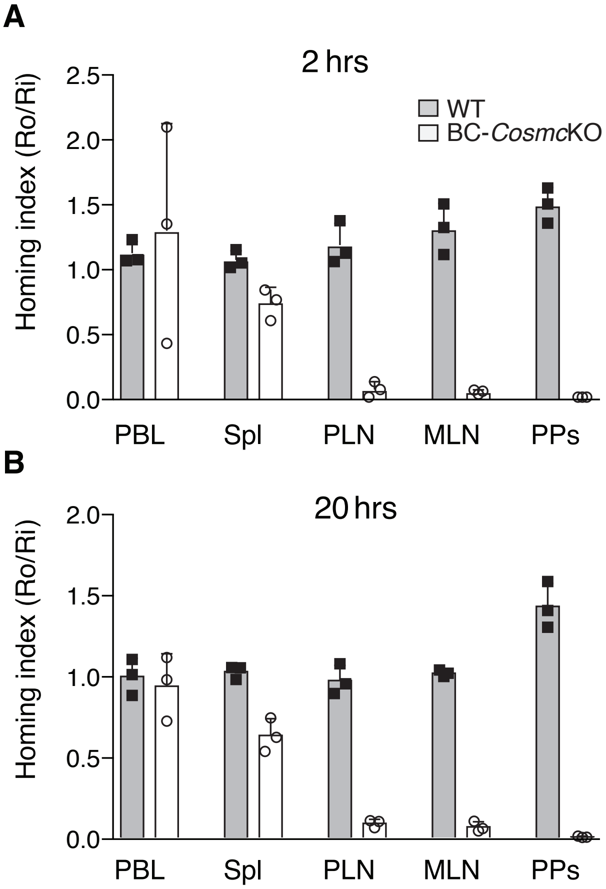

Supplementary Figure 2 Blocked homing of Cosmc-deficient B cells in BC-*Cosmc*KO mice. Splenic cells from WT and BC-*Cosmc*KO mice were labeled with CellTrace Violet dye separately and transferred to BC-*Cosmc*KO recipient mice (n=3 for both WT and BC-*Cosmc*KO donor cells) with CSFE labelled internal control. (A and B) Homed donor Tn^- or Tn^+ $B220^+$ (or $CD19^+$) B cells were harvested at 2 (A) or 20 (B) hours and analyzed by flow cytometry. The homing index was calculated as the [percentage of dye^+ Tn^- or Tn^+ B cells]$_{tissue}$/ [percentage of internal control dye^+ B cells]$_{tissue}$ ratio to the input ratio. Each symbol (black square and open circle for WT and BC-*Cosmc*KO, respectively) represents an individual mouse. Data are presented as average ±SD of each genotype. Source data are provided as a Source Data file.

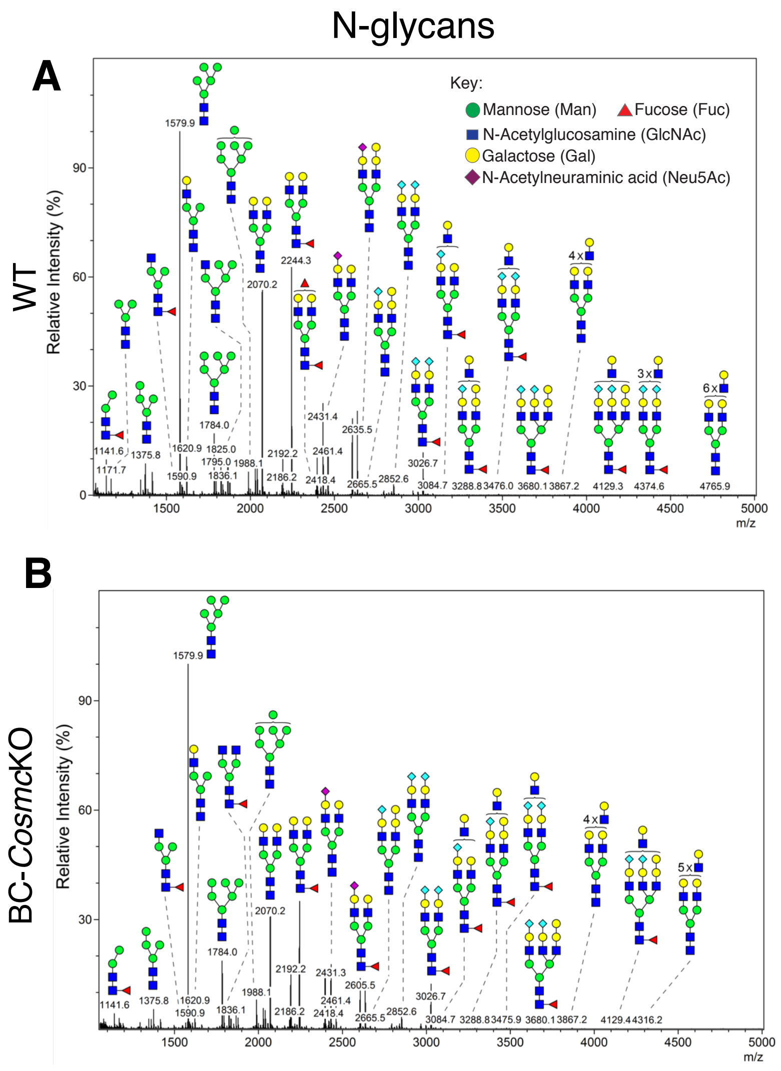

Supplementary Figure 3 N-glycan profiling of B cells. (A and B) N-Glycans were extracted from splenic B cells that were purified from WT (n=3) (A) and BC-*CosmcKO* (n=3) mice (B). The released glycans were subjected to mass spectrometric analysis. Source data are provided as a Source Data file.

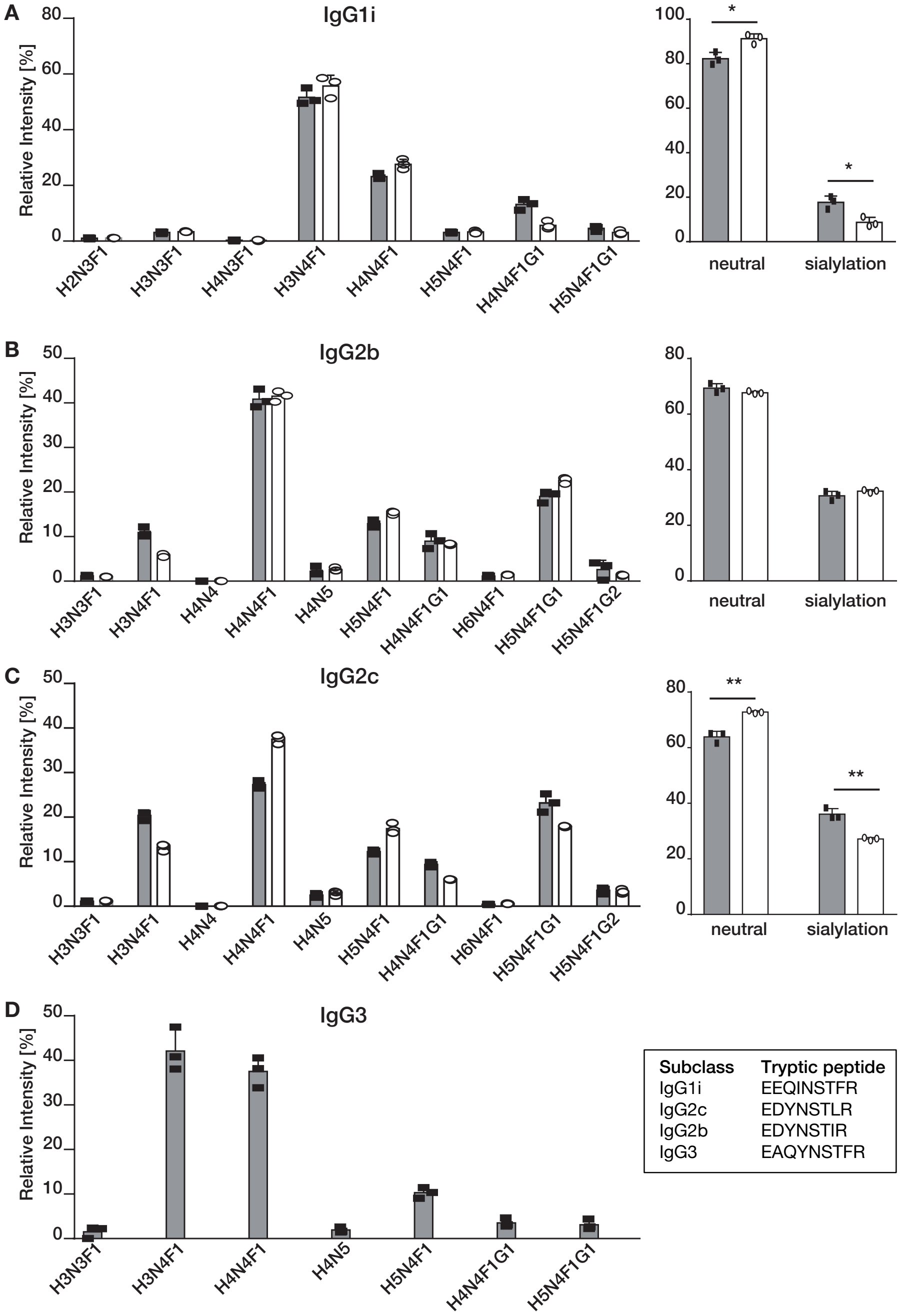

Supplementary Figure 4 Relative quantitation of WT and BC-CosmcKO IgG C_H2 domain N-glycopeptides in a subclass-dependent manner. (A) IgG1i N-glycopeptides with tryptic peptide sequence EEQINSTFR. p value=0.0123 for both groups. (B) IgG2b N-glycopeptides with tryptic peptide sequence EDYNSTIR. p value=0.1634 for both groups. (C) IgG2c N-glycopeptides with tryptic peptide sequence EDYNSTLR. p value=0.0017 for both groups. (D) IgG3 N-glycopeptides with tryptic peptide sequence EAQYNSTFR. IgG3 N-glycopeptides in the BC-CosmcKO serum were not quantified due to low signal intensities. For (A-C), the corresponding inserts show the comparison of neutral and sialylated N-glycans between both sample types. KO mice sera contained less IgG1i and IgG2c sialylation compared to WT. For (A-D), sera were collected from both WT (n=3) and BC-CosmcKO (n=3) mice at 8 weeks old. Each symbol (black square and open circle for WT and BC-CosmcKO, respectively) represents an individual mouse. Data are presented as average ±SD of each genotype. Unpaired two-tailed Student t tests were performed to determine statistical significance with ** $p<0.01$, * $p<0.05$. Glycan compositions are indicated: H–hexose; N–N-acetylhexosamine; F–fucose; G–N-glycolylneuraminic acid. Source data are provided as a Source Data file.

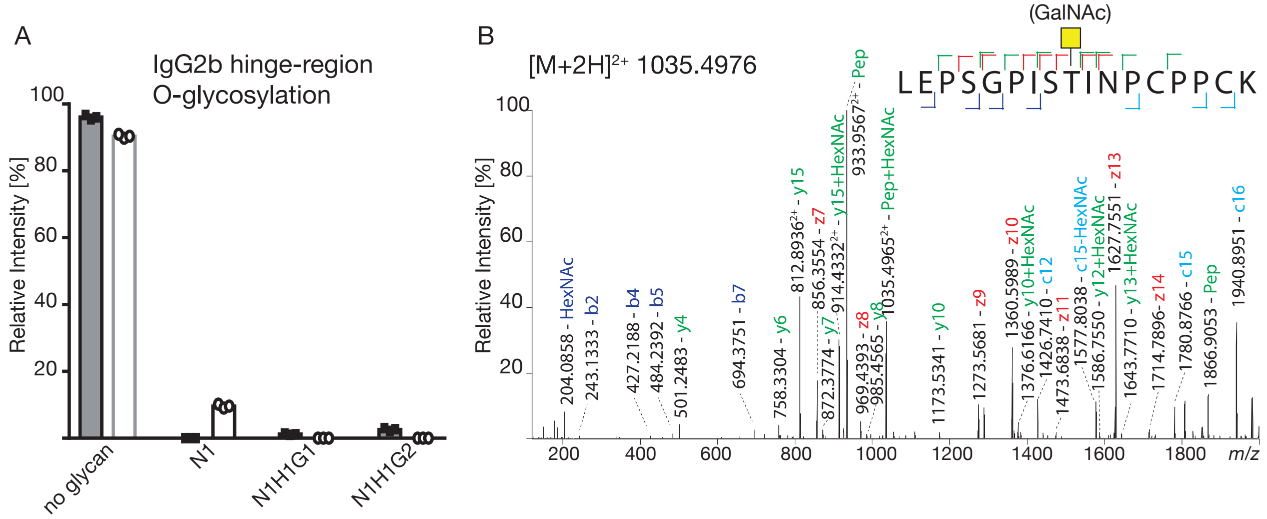

Supplementary Figure 5 IgG2b hinge-region O-glycosylation characterization in WT and BC-*Cosmc*KO serum. (A) The O-glycan distribution of the IgG2b hinge-region tryptic peptide LEPSGPISTINPCPPCK and the missed-cleaved peptide KLEPSGPISTINPCPPCK. Relative intensities were averaged for both peptide species. The majority of the hinge-region is unglycosylated. (B) EThcD spectra of the BC-*Cosmc*KO peptide LEPSGPISTINPCPPCK carrying a single GalNAc. For (A-B), sera were collected from both WT (n=3) and BC-*Cosmc*KO (n=3) mice at 8 weeks old. Each symbol (black square and open circle for WT and BC-*Cosmc*KO, respectively) represents an individual mouse. Data are presented as average ±SD of each genotype. Source data are provided as a Source Data file.

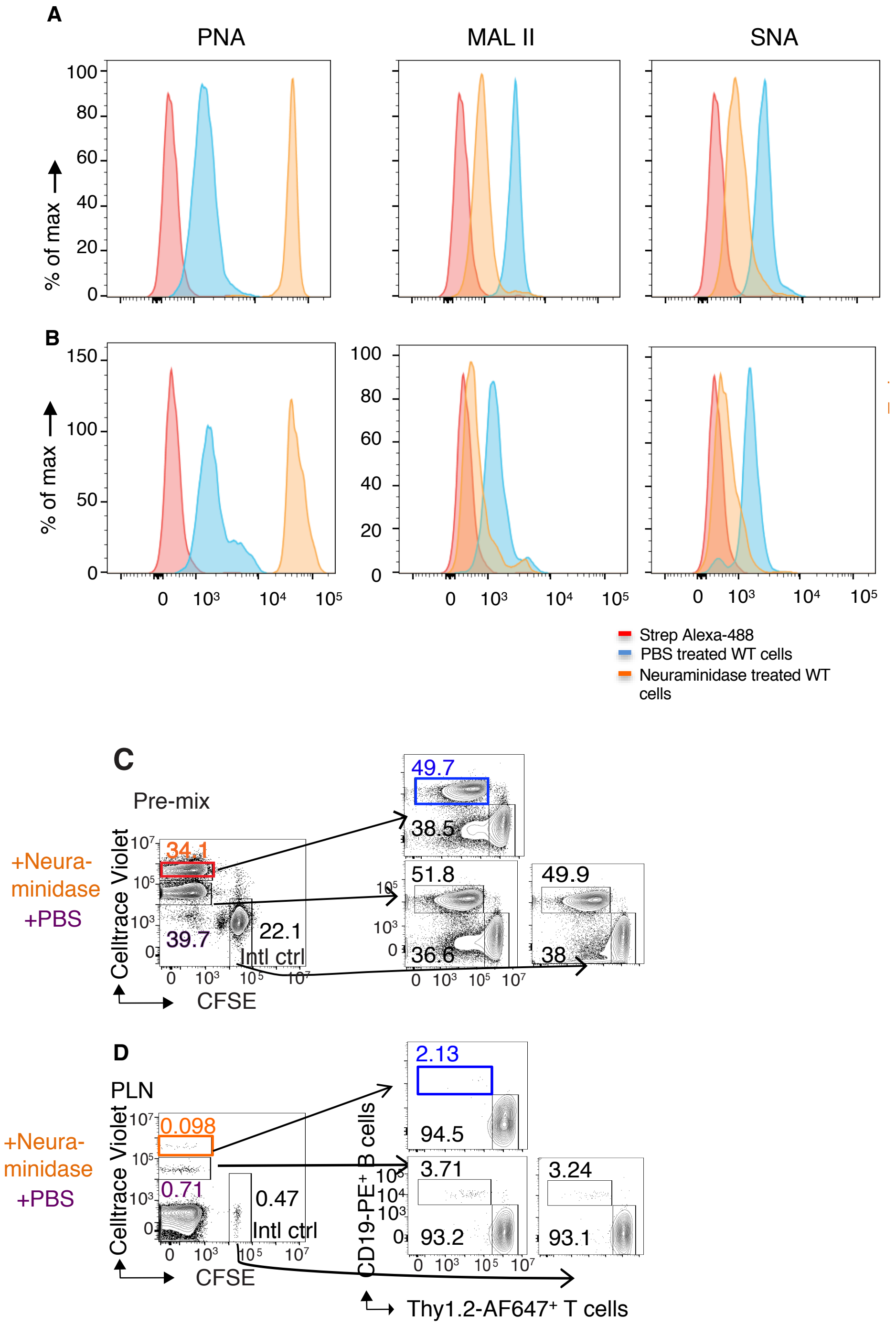

Supplementary Figure 6 Blocked homing of neuraminidase treated lymphocytes to lymph nodes. (A-B) Binding analysis of biotinylated PNA, SNA, and MAL-II, followed by streptavidin-Alexa 488, on neuraminidase or PBS treated WT splenic $CD19^{+}$ B cells (A) and $Thy1.2^{+}$ T cells (B). Histogram in pink: streptavidin-Alexa 488 alone as background control. Histogram in blue: cells treated with PBS. Histogram in yellow: cells treated with neuraminidase. (C-D) Splenocytes in single suspension from WT mice labeled with CellTrace Violet or CFSE dye and treated with neuraminidase (+Neur) and co-injected into recipient mice. Lymphoid tissues harvested at 1 hr after transfer. Representative flow cytometric dot plots show input cells ratio (C) before injection and after transfer collected from recipient peripheral lymph node (D). Neur in orange rectangle, and numbers in orange (percentage of total input): donor cells treated with Neur. PBS in purple numbers (percentage of total input): donor cells treated with PBS. Blue rectangle, and numbers in blue: Neur-treated B cells in panel C, and Neur-treated and transferred B cell population recovered from indicated tissue of recipients in panel D.

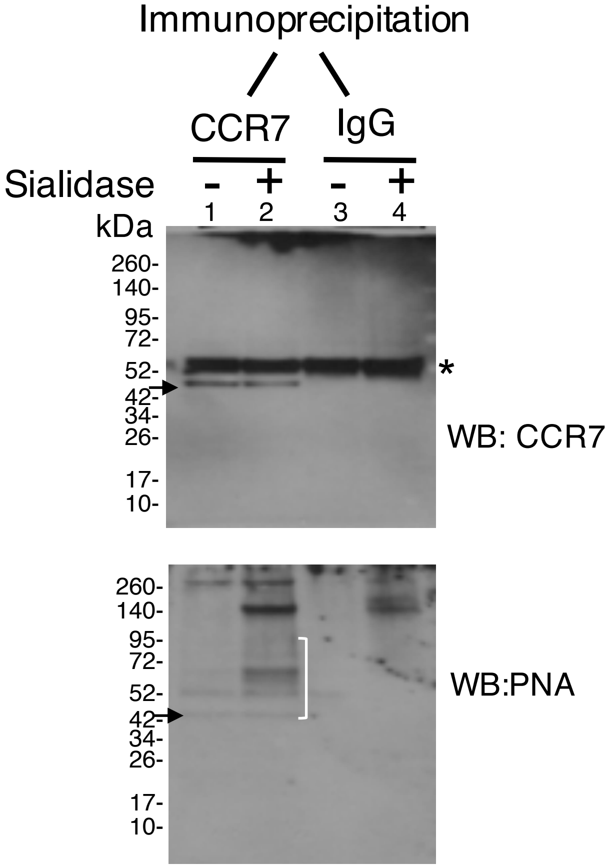

Supplementary Figure 7 CCR7 contains O-glycans. Splenocyte cell extracts from WT mice (n=3 animals from one experiment) were immunoprecipitated with anti-CCR7 or control IgG followed by treatment with or without sialidase; the immunoprecipitated materials were analyzed by SDS-PAGE followed by Western blot. Immunoblots were probed for CCR7 (Upper panel) and for core 1 O-glycan-containing glycoproteins with the plant lectin PNA (Bottom panel). CCR7 immunoblot shows the expected size for CCR7 in the pull down indicated by the black arrow (left). No significant material was present in the pull down with control antibody, as expected. * identifies antibodies used in pull down experiment bound by secondary anti-IgG reagents. Bottom, Western blotting with PNA shows staining of CCR7 bands. Interestingly, other PNA-reactive glycoproteins, possibly associated with CCR7 were also present in the anti-CCR7 pull down and stained with PNA after sialidase, as highlighted by the bracketed material in lane 2. Source data are provided as a Source Data file.

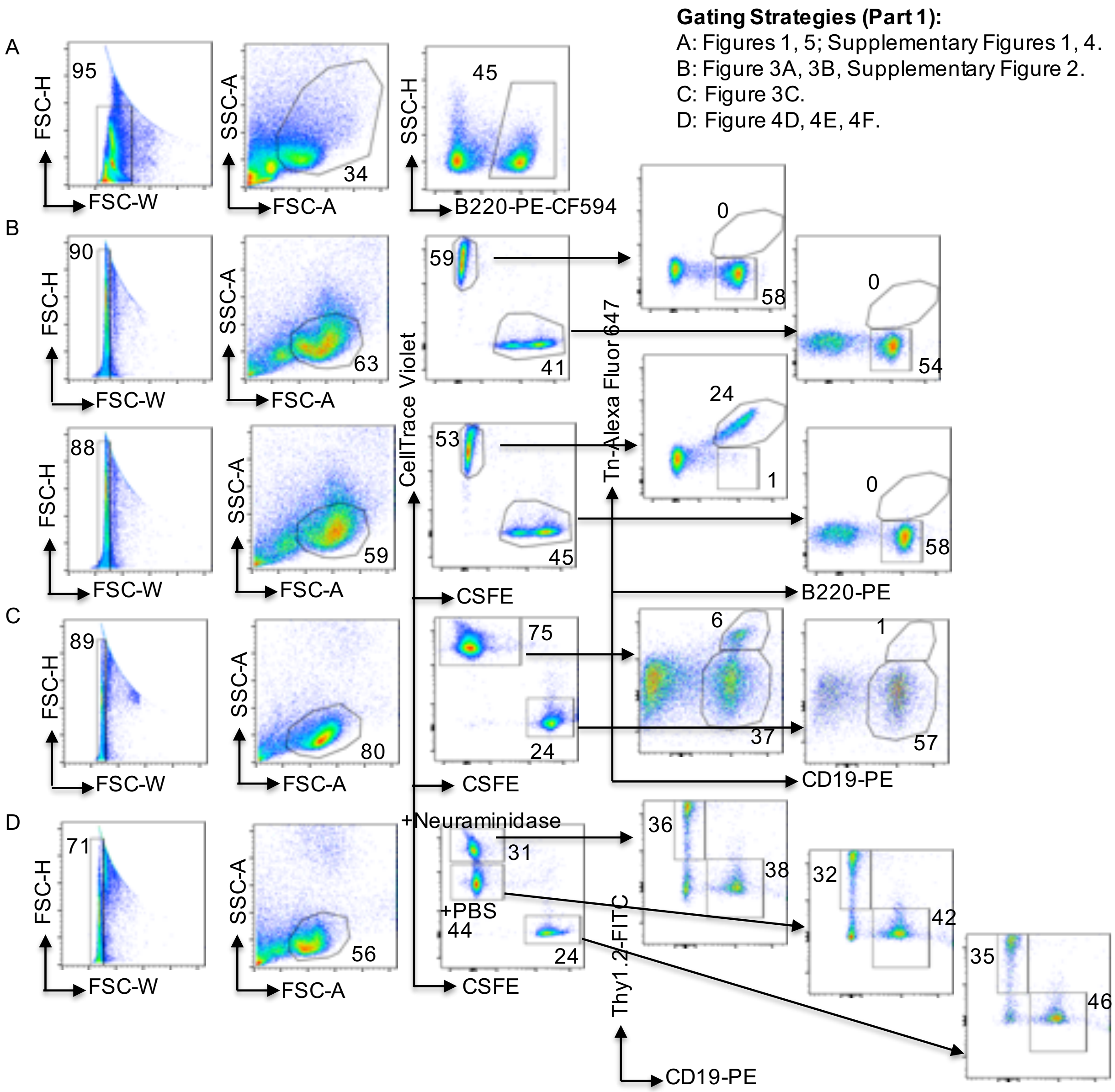

Supplementary Figure 8 Gating Strategies for Flow Cytometry (Part 1). (A) Gating strategy and plots used to generate data in Figures 1 and 5, and Supplementary Figures 1 and 4. (B) Gating strategy and plots used to generate data in Figures 3A and 3B, and Supplementary Figure 2. (C) Gating strategy and plots used to generate data in Figure 3C. (D) Gating strategy and plots used to generate data in Figure 4D, 4E, 4F. Source data are provided as a Source Data file.

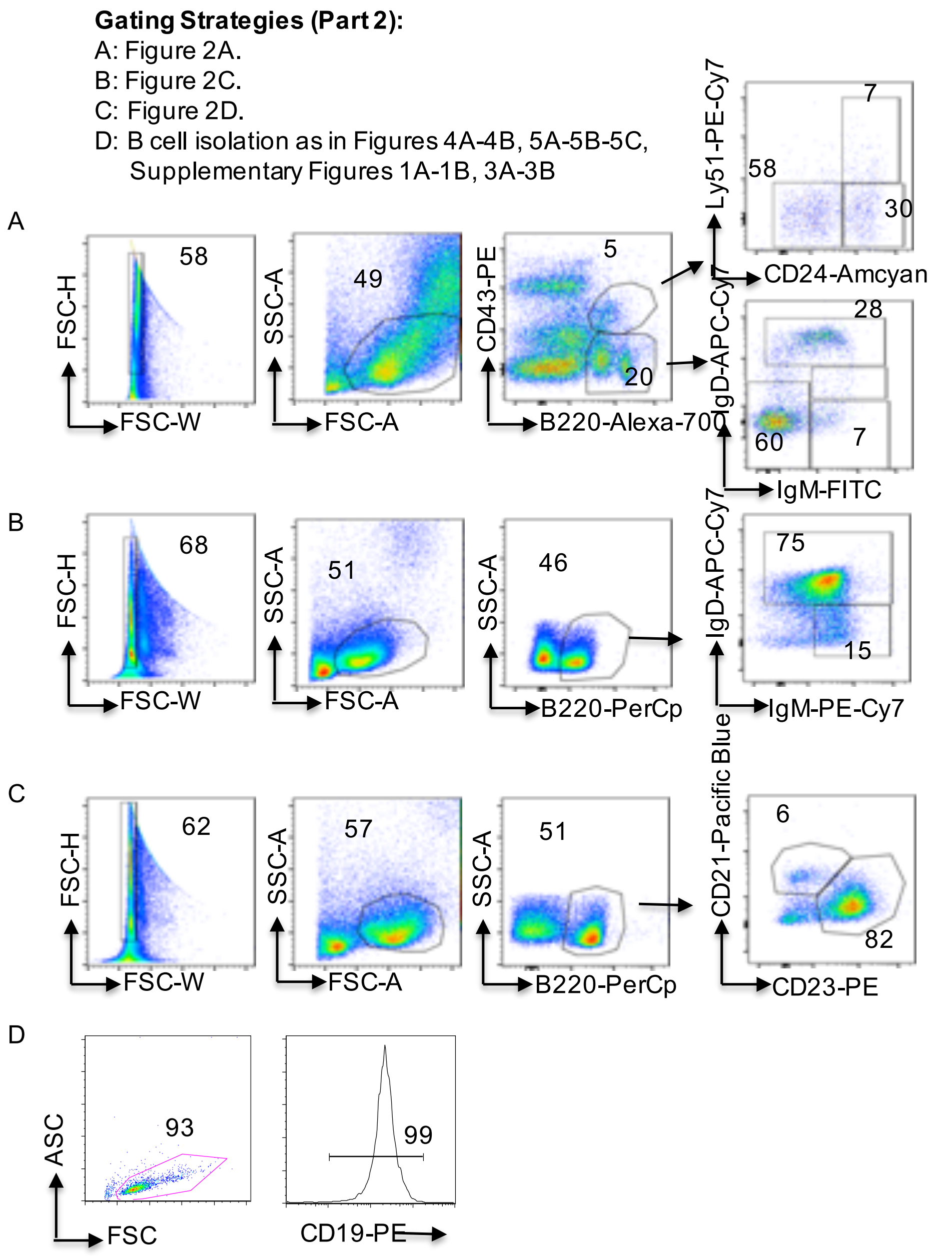

Supplementary Figure 9 Gating Strategies for Flow Cytometry (Part 2). (A) Gating strategy and plots used to generate data in Figure 2A. (B) Gating strategy and plots used to generate data in Figure 2C. (C) Gating strategy and plots used to generate data in Figure 2D. (D) Gating strategy and plots used B cell isolation in Figures 4A, 4B, 5A, 5B, 5C, and Supplementary Figures 1A, 1B, 3A, 3B. Source data are provided as a Source Data file.
