# Supplementary material for: Cosmc controls B cell homing
Source: Nat Commun. 2020 Aug 10;11:3990. doi: 10.1038/s41467-020-17765-6 (PMC7417590; doi:10.1038/s41467-020-17765-6)
Supplement: Supplementary file 3 — Reporting Summary [file 41467_2020_17765_MOESM3_ESM.pdf]

nature research

Corresponding author(s): Richard Cummings

Last updated by author(s): 07-01-2020

Reporting Summary

Nature Research wishes to improve the reproducibility of the work that we publish. This form provides structure for consistency and transparency in reporting. For further information on Nature Research policies, see our Editorial Policies and the Editorial Policy Checklist.

Statistics

For all statistical analyses, confirm that the following items are present in the figure legend, table legend, main text, or Methods section.

n/a	Confirmed	
☐	☒	The exact sample size (*n*) for each experimental group/condition, given as a discrete number and unit of measurement
☐	☒	A statement on whether measurements were taken from distinct samples or whether the same sample was measured repeatedly
☐	☒	The statistical test(s) used AND whether they are one- or two-sided<br>*Only common tests should be described solely by name; describe more complex techniques in the Methods section.*
☒	☐	A description of all covariates tested
☒	☐	A description of any assumptions or corrections, such as tests of normality and adjustment for multiple comparisons
☐	☒	A full description of the statistical parameters including central tendency (e.g. means) or other basic estimates (e.g. regression coefficient) AND variation (e.g. standard deviation) or associated estimates of uncertainty (e.g. confidence intervals)
☐	☒	For null hypothesis testing, the test statistic (e.g. *F*, *t*, *r*) with confidence intervals, effect sizes, degrees of freedom and *P* value noted<br>*Give P values as exact values whenever suitable.*
☒	☐	For Bayesian analysis, information on the choice of priors and Markov chain Monte Carlo settings
☒	☐	For hierarchical and complex designs, identification of the appropriate level for tests and full reporting of outcomes
☒	☐	Estimates of effect sizes (e.g. Cohen's *d*, Pearson's *r*), indicating how they were calculated

Our web collection on statistics for biologists contains articles on many of the points above.

Software and code

Policy information about availability of computer code

Data collection	BD: FACSDiva V.8.0.2/CytExpert V.2.3/CellQuest Pro5.2.1. Thermo Scientific: Multiskan Spectrum Perkin Elmer: Victor MultipleLabel Counter
Data analysis	FlowJo V.9 and 10 /Prism V.6/7. Image J (Fiji)

For manuscripts utilizing custom algorithms or software that are central to the research but not yet described in published literature, software must be made available to editors and reviewers. We strongly encourage code deposition in a community repository (e.g. GitHub). See the Nature Research guidelines for submitting code & software for further information.

Data

Policy information about availability of data

All manuscripts must include a data availability statement. This statement should provide the following information, where applicable:

- Accession codes, unique identifiers, or web links for publicly available datasets
- A list of figures that have associated raw data
- A description of any restrictions on data availability

All raw data in this study are available from the corresponding author upon reasonable request, and no restrictions on data availability.

Field-specific reporting

Life sciences study design

All studies must disclose on these points even when the disclosure is negative.

Sample size	No sample size calculation was performed in this study. Experiments were carried out independently in an unbiased manner with similar results. Sample size were determined based on experimental feasibility and results consistency, attempting to have a minimal of n=3 biological replicates or experiments with sufficient reproducibility. Exceptions included in supplementary Fig.1B, where Tsynthase activity was measured from B cells of 4WT and 4KO mice in total, in two independent experiments, and a representative experiment was displayed; α-mannosidase activity was measured from two mice of both groups. The purpose of the enzyme assays was to corroborate the conclusion drawn from RT-PCR (supplementary Fig.1A), flow cytometry (supplementary Fig.1C), that efficient deletion of Cosmc, and least effect on others, in B cells. The consistent results of similar from 2WT and 2KO mice already provide enough confidence. Likewise, the consistent results of Fig.1F and supplementary Fig.2 also provide enough confidence to draw conclusions and minimize the use of mice.
Data exclusions	No data were excluded from the analyses.
Replication	All experimental findings were repeated at least 3 times with sufficient reproducibility, except for those mentioned in sample size.
Randomization	Animals were allocated randomly with matched age and gender.
Blinding	Group allocation was not blinded to investigators during data acquisition and analyses because the KO mice are maintained in the same cage as the WT littermates. Genotyping was performed beforehand so the investigator can plan the experiments and minimize the use of animals.

Reporting for specific materials, systems and methods

We require information from authors about some types of materials, experimental systems and methods used in many studies. Here, indicate whether each material, system or method listed is relevant to your study. If you are not sure if a list item applies to your research, read the appropriate section before selecting a response.

Materials & experimental systems

n/a	Involved in the study	
☐	☒	Antibodies
☒	☐	Eukaryotic cell lines
☒	☐	Palaeontology and archaeology
☐	☒	Animals and other organisms
☒	☐	Human research participants
☒	☐	Clinical data
☒	☐	Dual use research of concern

Methods

n/a	Involved in the study	
☒	☐	ChIP-seq
☐	☒	Flow cytometry
☒	☐	MRI-based neuroimaging

Antibodies

Antibodies used	Antibodies from BD Pharmingen: Thy1.2-FITC, clone#30-H12,cat#553013; CD19-PE, clone#1D3,cat#553786; B220-PE/PE-CF594/PerCP, clone#RA3-6B2,cat#553090/562290/553093; CD62L (L-selectin)-FITC, clone#MEL14, cat#553150; β7-PE, clone#M293,cat#557498, CD23-PE, clone#B3B4, cat#553139, IgM-FITC, clone#II/41, cat#553437, CD43-PE, clone#S7, cat#561857.<br>Antibodies from Biolegend: Thy1.2-AF647, clone#30-H12, cat#105318, IgM-PE-Cy7, clone#RMM-1, cat#406514, IgD-APC-Cy7, clone#11-26c.2a, cat#405716, CD21-Pacific Blue, clone#123414, cat# 7E9, B220-Alexa Fluor-700, clone#RA3-6B2, cat#103232, CD24-BV510, clone#M1/69, cat#101831, Ly51-PE-Cy7, clone#6C3, cat#108313, α4β7-PE, clone#DATK32, cat#120605 (also from eBbioscience,same clone, cat#12-5887-81); CXCR5-PE-Cy7, clone#L138D7, cat#145515; CCR7-FITC, clone#4B12, cat#120112; CXCR4-PE, clone#L276F12, cat#146505.<br>Streptavidin-Alexa Fluor 488 from Life technologies, cat#S32354.<br>Tn-Alexa Fluor 647 was prepared in the lab.<br>Antibodies for ELISA, from Southern Biotech: mouse antibody standards (IgG1, clone#15H6, cat#0102-01, IgG2b, clone#A-1, cat#0104-01, IgG2c, clone#6.3, cat#0122-01, IgG3, clone#B10, cat#0105-01, IgM, clone#11E10, cat#0101-01, IgA, clone#S107, cat#0106-01), Unconjugated goat anti-mouse antibodies (IgG1, cat#1071-01, IgG2b, cat#1091-01, IgG2c, cat#1078-01, IgG3, cat#1101-01, IgM, cat#1020-01, IgA, cat#1040-01), HRP-conjugated antibodies(IgG1, cat#1070-05, IgG2b, cat#1090-05, IgG2c, cat#1079-05, IgG3, cat#1100-05, from Life Technologies: IgM, cat#M31507, IgA, cat#62-6720).<br>Biotinylated lectins are from Vector Laboratories: Biotinylated Peanut Agglutinin (PNA), cat#B-1075; Biotinylated Sambucus nigra Lectin (SNA), cat#B-1305; Biotinylated Maackia amurensis Lectin II (MAL II), cat#B-1265.
Validation	All primary antibodies, except for anti-Tn antibody, and lectins were commercially available and their reactivities and applications were validated by the providers and according statements are available on the provider's website.

Animals and other organisms

Policy information about studies involving animals; ARRIVE guidelines recommended for reporting animal research

Laboratory animals	2-month old male mice with C57 BL/6 background were used in all experiments.
Wild animals	No wild animals were involved.
Field-collected samples	No field-collected samples.
Ethics oversight	All experiments involving mice were performed in accordance with approved protocols from the Institutional Animal Care and Use Committee (IACUC) at Beth Israel Deaconess Medical Center, Harvard Medical School, and the approved protocols were strictly followed.

Note that full information on the approval of the study protocol must also be provided in the manuscript.

Flow Cytometry

Plots

Confirm that:

- [x] The axis labels state the marker and fluorochrome used (e.g. CD4-FITC).
- [x] The axis scales are clearly visible. Include numbers along axes only for bottom left plot of group (a 'group' is an analysis of identical markers).
- [x] All plots are contour plots with outliers or pseudocolor plots.
- [x] A numerical value for number of cells or percentage (with statistics) is provided.

Methodology

Sample preparation	Tissues were collected and dissociated through a cell strainer with a plunger of a 3 cc syringe.
Instrument	BD LSR II, Calibur, and Cytoflex LX.
Software	FlowJo V.9 and 10 were used to analyze the data
Cell population abundance	B cell isolation kit (Miltenyi Biotech # 130-090-862) was used to isolate B cells. Purity of isolated spleen B cells is equal to or greater than 92%, and determined by CD19 positivity by flow cytometry.
Gating strategy	Cells were gated on lymphocyte gating, and doublets were excluded, if applicable.

- [x] Tick this box to confirm that a figure exemplifying the gating strategy is provided in the Supplementary Information.
