# Supplementary material for: Cosmc controls B cell homing
Source: Nat Commun. 2020 Aug 10;11:3990. doi: 10.1038/s41467-020-17765-6 (PMC7417590; doi:10.1038/s41467-020-17765-6)
Supplement: Supplementary file 4 — Description of Additional Supplementary Files [file 41467_2020_17765_MOESM4_ESM.pdf]

Description of Additional Supplementary Files

Supplementary Movie 1: IVM of WT B cells. Calcein-labelled WT B cells were injected in the left femoral artery and IVM of the right inguinal lymph node was conducted in an anesthetized WT mouse. Adoptively transferred cells appear as bright dots within blood vessels (not visible). The size of the field of view is 1,339 µm x 1,331 µm.

Supplementary Movie 2: IVM of BC-*Cosmc*KO B cells. Calcein-labelled BC-*Cosmc*-KO B cells were injected in the left femoral artery and IVM of the right inguinal lymph node was conducted in an anesthetized WT mouse. Adoptively transferred cells appear as bright dots within blood vessels (not visible). The size of the field of view is 1,339 µm x 1,331 µm.
